# The Economic Burden of Influenza-Like Illness among Children, Chronic Disease Patients, and the Elderly in China: A National Cross-Sectional Survey

**DOI:** 10.3390/ijerph18126277

**Published:** 2021-06-10

**Authors:** Xiaozhen Lai, Hongguo Rong, Xiaochen Ma, Zhiyuan Hou, Shunping Li, Rize Jing, Haijun Zhang, Yun Lyu, Jiahao Wang, Huangyufei Feng, Zhibin Peng, Luzhao Feng, Hai Fang

**Affiliations:** 1 School of Public Health, Peking University, Beijing 100083, China; laixiaozhen@pku.edu.cn (X.L.); rzjing2015@hsc.pku.edu.cn (R.J.); zhanghj966@bjmu.edu.cn (H.Z.); lydialu1217@hotmail.com (Y.L.); 1510306210@pku.edu.cn (J.W.); yffenghuang@pku.edu.cn (H.F.); 2 China Center for Health Development Studies, Peking University, Beijing 100083, China; hgrong@hsc.pku.edu.cn (H.R.); xma@hsc.pku.edu.cn (X.M.); 3 Institute for Excellence in Evidence-Based Chinese Medicine, Beijing University of Chinese Medicine, Beijing 100029, China; 4 School of Public Health, Fudan University, Shanghai 200032, China; zyhou@fudan.edu.cn; 5 School of Health Care Management, Cheeloo College of Medicine, Shandong University, Jinan 250012, China; lishunping@sdu.edu.cn; 6 NHC Key Laboratory of Health Economics and Policy Research (Shandong University), Jinan 250012, China; 7 Division of Infectious Diseases, Chinese Center for Disease Control and Prevention, Beijing 102206, China; pengzb@chinacdc.cn; 8 School of Population Medicine and Public Health, Chinese Academy of Medical Sciences & Peking Union Medical College, Beijing 100730, China; 9 Peking University Health Science Center—Chinese Center for Disease Control and Prevention Joint Center for Vaccine Economics, Peking University, Beijing 100083, China; 10 Key Laboratory of Reproductive Health, National Health Commission of the People’s Republic of China, Beijing 100083, China

**Keywords:** economic burden, influenza-like illness, healthcare-seeking behaviors, China

## Abstract

**Background:** The disease burden of seasonal influenza is substantial in China, while there is still a lack of nationwide economic burden estimates. This study aims to examine influenza-like illness (ILI) prevalence, healthcare-seeking behaviors, economic impact of ILI, and its influencing factors among three priority groups during the 2018–19 influenza season. **Methods:** From August to October 2019, 6668 children’s caregivers, 1735 chronic disease patients, and 3849 elderly people were recruited from 10 provinces in China to participate in an on-site survey. The economic burden of ILI consisted of direct (medical or non-medical) and indirect burdens, and a two-part model was adopted to predict the influencing factors of total economic burden. **Results:** There were 45.73% children, 16.77% chronic disease patients, and 12.70% elderly people reporting ILI, and most participants chose outpatient service or over-the-counter (OTC) medication after ILI. The average economic burden was CNY 1647 (USD 237.2) for children, CNY 951 (USD 136.9) for chronic disease patients, and CNY 1796 (USD 258.6) for the elderly. Two-part regression showed that age, gender, whether the only child in the family, region, and household income were important predictors of ILI economic burden among children, while age, region, place of residence, basic health insurance, and household income were significant predictors of ILI economic burden among chronic disease patients and the elderly. **Conclusions:** A large economic burden of ILI was highlighted, especially among the elderly with less income and larger medical burdens, as well as children, with higher prevalence and higher self-payment ratio. It is important to adopt targeted interventions for high-risk groups, and this study can help national-level decision-making on the introduction of influenza vaccination as a public health project.

## 1. Introduction

Annual influenza epidemics result in substantial morbidity and mortality across the globe, with a large share of the total disease burden occurring in low- and middle-income countries (LMICs) [1]. As estimated by the World Health Organization (WHO) in 2018, the annual epidemics of seasonal influenza caused 3–5 million severe cases and 290,000–650,000 deaths [2]. The disease burden of seasonal influenza is also substantial in mainland China, with about 88,000 excess deaths occurring annually [3]. Illness severity and mortality are the greatest in high-risk groups, and so are the associated healthcare costs and productivity losses.

Immunization has been proven to be one of the most cost-effective health investments to prevent and control influenza, with strong positive externalities [4,5,6], so public intervention is expected to drive vaccine coverage to a socially optimal level [7]. The WHO and Chinese Center for Disease Control and Prevention recommended that children, chronic diseases patients, and the elderly be among the priority groups for influenza vaccination [8,9]. However, influenza vaccination has not been included in China’s National Immunization Program (NIP), and the expenses are paid out of pocket on most occasions, even for priority groups. The coverage rate of influenza vaccination in China has been extremely low in the past 15 years, with only 2% of the entire population being immunized, and small-scale policy interventions have failed to increase national uptake of influenza vaccinations [10].

As the WHO reported, as of the end of 2019, 119 out of 194 member states had included influenza vaccination in their NIPs [11], and the economic burden could decrease to a large extent after the introduction of vaccines in NIP. In the United States, a study published early in 2007 estimated the medical and indirect costs attributable to annual influenza epidemics, and found that the total economic burden amounted to USD 87.1 billion annually [12]. More recently, in 2018, researchers provided an updated estimate for the United States after vaccination efforts, and found a substantially lower (approximately half) total cost than previously estimated [13]. By comparison, China has not introduced influenza vaccination into its NIP, so it is even more important to estimate the economic burden of influenza in China to promote decision-making.

Access, affordability, and equity are three basic goals of a well-operated health system [14], so evidence of the economic burden of influenza can help raise awareness in the public and clinical communities of the burden and consequences of this important disease. Additionally, estimating the economic burden of influenza is crucial to support national-level decision-making, while sufficient data to precisely estimate the economic burden of influenza are scarce and incomplete in LMICs [15]. In China, most previous studies were conducted in developed southern areas at the hospital or provincial level, and the economic burden was not evaluated in an all-sided way. Besides, little research was done among different priority groups to make horizontal comparisons, and there also was a lack of national-level community-based estimates of influenza-like illness (ILI) prevalence and healthcare-seeking behaviors.

To address these gaps, the present study collected individual data in 10 provinces in China to examine ILI prevalence in communities, influenza-related healthcare-seeking behaviors, the economic impact of ILI, and its influencing factors among children, chronic disease patients and the elderly during the 2018–2019 influenza season. Among the objectives, the primary goal was to calculate the economic impact of ILI for three priority groups in a national-level community-based survey. We hypothesized that the ILI prevalence, healthcare-seeking behaviors, ILI economic burden, and its composition would vary across different priority groups, and that many sociodemographic factors would have an influence on the economic burden of ILI, such as age, gender, and income. We also paid attention to regional and rural–urban differences in ILI economic burdens, and we hypothesized that patients living in less-developed areas (rural and western) would have a lower economic burden, given the lower lost productivity and direct expenditures [16].

The present study is organized as follows. The first part provides the background information and objectives of the study; the second part presents a literature review on the economic burden of influenza in China; the third part elaborates on the materials and methods adopted in the present study; the fourth part displays the statistical description and study results; the fifth part discusses the results, gives some policy implications, and puts forward the limitations of this study; and the sixth part briefly summarizes the present study.

## 2. Literature Review

Influenza can impose a substantial socioeconomic burden on families and society. Table 1 summarizes the studies conducted in China that explored the economic burden of influenza, but most of them were done at the hospital or provincial level, and direct non-medical costs or indirect costs were often missed [16,17,18,19,20]. In 2007, a household survey conducted in Guangdong Province found that the cost for an ILI episode was about one-fifth of monthly per capita income, but the study did not ask for the direct non-medical costs of respondents [16]. Similarly, a prospective study conducted in Zhuhai City during 2008–2009 found that direct medical costs of influenza created a substantial economic burden in the outpatient setting, but it did not examine the direct non-medical costs and family labor losses [17]. In Shanghai, a study estimated only the direct medical costs of influenza among the elderly aged over 60 years, and found that the average outpatient and inpatient direct costs were USD 47 and USD 1601, respectively [18]. In Suzhou, two studies were performed to explore the economic burden of influenza in outpatient and inpatient settings, respectively, and both of them focused on children aged less than 5 years [19,20]. The former reported the healthcare-seeking behaviors of outpatients, and collected non-medical or indirect costs via telephone surveys. It found that influenza would impose a heavy economic burden on children’s families [19]. The latter collected medical costs and hospital length of stay for pneumonia and influenza inpatients, but did not access non-medical or indirect costs [20].

Besides those at the hospital or provincial level, there were two cross-province studies on the economic burden of influenza [21,22]. One examined the direct medical cost of influenza-related hospitalizations in three provinces, in which non-medical or indirect costs were not examined [21]. The other was a telephone survey in 10 provinces to collect costs in an all-sided way, but it was conducted among the general population [22]. Overall, there were some studies concerning the economic burden of ILI or influenza in China, and they could help in developing influenza control policies. However, most of them were conducted in developed southern China, and did not report the healthcare-seeking behaviors, non-medical costs, indirect costs, or over-the-counter (OTC) medication costs of targeted high-risk groups. In this case, we conducted this survey, trying to fill these gaps.

## 3. Materials and Methods

### 3.1. Study Population and Sampling

In August to October 2019, a total of 12,252 participants in 148 community health centers from 10 provinces in China were approached to join the national survey on the economic burden of seasonal influenza, including 6668 children aged 6–59 months, 1735 chronic disease patients aged 18–59 years, and 3849 elderly people aged above 60 years (Financing Strategies of Influenza Vaccination in China, NCT04038333) [23]. For children aged 6–59 months, we asked their parents or grandparents who accompanied them to health centers to finish the compulsory immunization procedure [9]. For chronic disease patients and the elderly, we asked them in health centers or gathered them in neighborhood committees. This study was ethically reviewed and approved by the Peking University Institutional Review Board (IRB00001052-19076), and written informed consent was obtained from individual or guardian participants.

**Figure 1:**
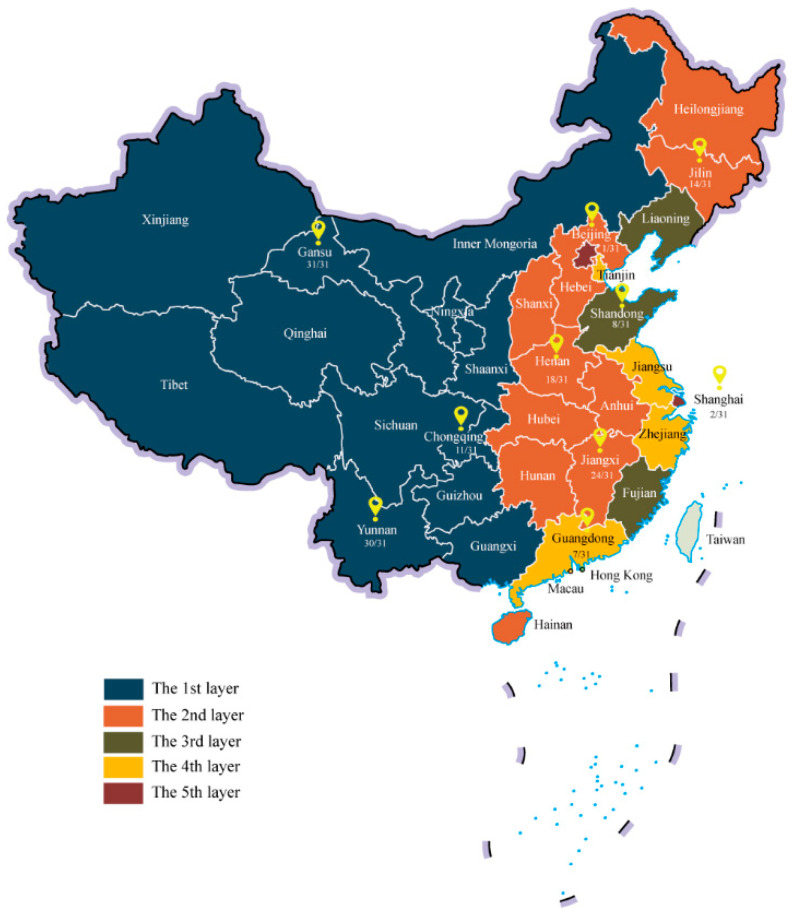
Ten provinces/municipalities selected for survey on the economic burden of influenza-like illness in China. In the survey, 10 provinces/municipalities (3, 3, 1, 1, and 2 in each layer) were chosen, including Beijing, Shanghai, Jilin, Yunnan, Shandong, Guangdong, Jiangxi, Gansu, Chongqing, and Henan. Their location and 2018 per capita GDP rank (e.g., 1/31) are marked in the figure.

The survey adopted a multistage sampling method. First, 10 provinces/municipalities were selected based on China’s Division of Central and Local Financial Governance and Expenditure Responsibilities in the Healthcare Sector, which stratifies the 31 provinces/municipalities into five layers [24]. In terms of location, socioeconomic development, and accessibility, 10 provinces/municipalities (3, 3, 1, 1, and 2 in each layer) were chosen, with their location and 2018 per capita GDP rank (e.g., 1/31) recorded in Figure 1. Second, in each province/municipality, a capital city or well-developed district and a non-capital city or less-developed district were selected. Third, two subdistricts/counties were chosen in each city or district, in which three or more immunization centers and the corresponding neighborhood committees were approached. The sample size was calculated under ILI prevalence assumptions, with an allowable error of 5% and disqualification rate of 10%.

### 3.2. Measures

The on-site survey was conducted by trained interviewers using a specially designed online questionnaire system on a portable Android device (PAD). The questionnaire was stored in advance in the PAD system, and interviewees would fill in the online questionnaire according to the answers of each respondent. Automatic logical proofreading was adopted in the online questionnaire to reduce input errors and missing values, and interview recordings were uploaded and spot-checked by quality-control personnel to find and correct problems in time. The structured online questionnaire collected: (1) sociodemographics; (2) whether the respondent had ILI in the past season; (3) healthcare-seeking behaviors after ILI; and (4) economic burden of the latest ILI in terms of direct (medical and non-medical) costs and indirect costs [25]. 

According to the WHO, body temperature ≥38 °C with either cough or sore throat was used in the present study to distinguish influenza from other respiratory illnesses [26]. For each interviewee (or their child aged 6–59 months) who reported ILI in the past season, direct medical costs were queried in terms of service type (outpatient service, inpatient service, and OTC medication) and co-payment in each service type; direct non-medical costs were inquired regarding aspects of transportation, nutrition/food, accommodation, and nursing-worker hiring; and indirect burden was obtained by multiplying the lost labor days of the respondents and their families and daily per capita household income of the respondents [25]. The reported direct medical and non-medical costs were adjusted for the provincial healthcare price index and consumer price index, respectively, according to the China Statistical Yearbook 2019 [27]. Total economic burden was calculated by summing up the adjusted values of direct medical cost, direct non-medical cost, and indirect cost for each respondent.

### 3.3. Statistical Analysis

We produced summary statistics of all respondents using frequencies and proportions for categorical variables, and means and standard deviations for continuous variables. The chi-square and Mann–Whitney tests were used to assess differences in sample characteristics. The economic burden values of ILI was displayed as means and 95% confidence intervals (CI) for those who reported ILI in the last influenza season.

In predicting the influencing factors of total economic burden, a two-part model was adopted because there were a large number of zeros in terms of total economic burden [28,29], and results are shown as coefficients and 95% CIs. The two-part model has been widely used in health-economics research [30,31]. In the first part of the regression, Y is a binary discrete dependent variable to estimate the probability of reporting ILI economic burden (if respondents report any economic burden of ILI, Z′ = 1; otherwise, Z′ = 0). We adopted the Probit model for regression and the maximum likelihood method for estimation, and the marginal effects of Part 1 are shown in Appendix A. The second part includes whether respondents truly received medical treatments and paid for them using ordinary least squares regression (If Z′ = 1, respondents will be included in the second part of regression). We also took the log form of total economic burden, which obeyed the skewed distribution to capture the nonlinear property of the association.

The indicators of the two-part model were chosen in the light of previous literature [32,33,34]. We took age (different age groups for the three priority populations), gender (male and female), whether the only child in the family (only for the children group), household monthly per capita income (CNY 1000; CNY 1 = USD 0.144 on 13 August 2020), place of residence (urban and rural), region (western, central, and eastern) and basic medical insurance type (medical insurance for urban and rural residents, urban employee medical insurance, and without basic medical insurance) as possible influencing factors. We also controlled self-reported health status (good, fair, or poor), but we did not display the results because they are not easily observable for policy-makers.

We further used multivariate Tobit regression concentrating on boundary value 0 for all respondents to predict the influencing factors of total economic burden (in log form), and the results are shown in Appendix B. A two-sided *p*-value below 0.05 was considered statistically significant in the present study. All data were analyzed using Stata version 14.0 (Stata Corp., College Station, TX, USA).

## 4. Results

### 4.1. Study Sample Characteristics

A total of 12,252 valid questionnaires (6668 for children, 1735 for patients with chronic diseases, and 3849 for the elderly) were received, with an effective response rate of 99.80%. Table 2 shows the general characteristics of participants. Overall, 45.73% of children, 16.77% of chronic disease patients, and 12.70% of elderly people reported to have ILI in the past season. Among children, older children aged 3–5 years had a higher possibility of catching ILI than younger children (*p* < 0.01), while the only child in the family (*p* < 0.01) and children living in western areas (*p* < 0.01) were less likely to have ILI than those living in families with more than one child and those living in central or eastern China. Among chronic disease patients, those with a younger age were more likely to catch ILI than older adults (*p* < 0.01). Among the elderly, respondents with higher household monthly per capita income (*p* < 0.05), living in urban areas (*p* < 0.01), living in eastern areas (*p* < 0.01), or having urban employee medical insurance (*p* < 0.01) had a lower possibility of having ILI. In the three groups, respondents with fair or poor self-reported health status had higher risk of catching ILI than those with good self-reported health status (*p* < 0.01). We further compared the distribution of gender and age among the three groups with that recorded in the China Population and Employment Statistics Yearbook 2019 [35], and found similar results, indicating the national representativeness of the population collected in this study.

### 4.2. Healthcare-Seeking Behaviors

Table 3 shows the distribution of healthcare-seeking behaviors among 3829 participants after the latest ILI by calculating the number of ILI cases leading to outpatient visits, hospitalization, and OTC medication. As the table indicates, most participants chose “Outpatient service only”, “OTC medication only”, or “Outpatient + OTC” after ILI, accounting for 84.29% of children, 84.53% of chronic disease patients, and 73.42% of elderly people.

More specifically, when ILI occurred in children, 38.11% of them only sought outpatient service, and 35.82% accepted a combination of outpatient treatment and OTC medication. There were also 102 children (3.35%) not receiving any medical services after ILI. In terms of service type, 83.96% of children received outpatient service after ILI, 12.36% had inpatient service, and 51.39% received OTC medication. As for chronic disease patients, 34.36% only sought outpatient service, and 26.46% received outpatient treatment and OTC medication. There were 20 chronic disease patients (6.87%) not receiving any medical services after ILI. In terms of service type, 67.70%, 8.59%, and 53.95% of chronic disease patients received outpatient service, inpatient service, and OTC medication, respectively. For the elderly, 32.52% of them only sought outpatient service, and 25.15% only received OTC medication. There were 32 elderly people (6.54%) not receiving any medical services. In terms of service type, 60.12%, 20.04%, and 47.44% of elderly people received outpatient service, inpatient service, and OTC medication, respectively.

### 4.3. Economic Burden of Influenza-Like Illness

Table 4 shows the economic burden of the three priority groups for the latest ILI episode during the 2018–2019 influenza season. As the results indicated, the economic burden of ILI for children was about CNY 1647, including reimbursed medical expenses of CNY 272, self-paid medical expenses of CNY 997, direct non-medical expenses of CNY 212, and indirect cost of CNY 166. The average economic burden for chronic disease patients was about CNY 951, including reimbursed medical expenses of CNY 335, self-paid medical expenses of CNY 386, direct non-medical expenses of CNY 102, and indirect cost of CNY 92. The elderly had a much higher average economic burden of about CNY 1796, including reimbursed medical expenses of CNY 766, self-paid medical expenses of CNY 778, direct non-medical expenses of CNY 162, and indirect cost of CNY 90. For children and chronic disease patients, outpatient and inpatient costs accounted for the vast majority of direct medical expenses, and nutrition/food and transportation costs accounted for most direct non-medical expenses. For the elderly, only inpatient costs accounted for the vast majority of direct medical expenses, and nutrition/food and accommodation costs accounted for most direct non-medical expenses.

### 4.4. Influencing Factors of Total Economic Burden

Table 5 shows the two-part model results to predict the influencing factors of total economic burden (in log form), including age, gender, whether the only child in the family (only for children), household income, place of residence, region, and basic medical insurance type (see Appendix A for marginal effects of Part 1, and see Appendix B for the results of the multivariate Tobit regression).

In the first part of the regression, we found that among children, the probability of an economic burden of ILI occurring was higher for older children aged 3–5 years (Coef. = 0.37, 95% CI 0.31–0.43, *p* < 0.05), boys (Coef. = 0.06, 95% CI 0.00–0.12, *p* < 0.05), and those living in central (Coef. = 0.50, 95% CI 0.42–0.58, *p* < 0.05) and eastern (Coef. = 0.40, 95% CI 0.32–0.48, *p* < 0.05) areas compared with younger children, girls, or those living in western China. Among chronic disease patients, those with older age tended to have a lower probability of having an ILI economic burden (*p* < 0.05) compared with younger respondents who developed chronic diseases at an earlier age. Among the elderly, those living in eastern areas (Coef. = −0.25, 95% CI −0.38 – −0.12, *p* < 0.05) and those with urban employee medical insurance (Coef. = −0.21, 95% CI −0.36 – −0.07, *p* < 0.05) were less likely to have an economic burden for ILI than those living in western China or those with medical insurance for urban and rural residents.

In the second part, patients with higher household monthly per capita income reported a larger economic burden for ILI than those with lower income in the three groups (*p* < 0.05). Among children, the economic burden of ILI was smaller for older children aged 3–5 years (Coef. = −0.16, 95% CI −0.26 – −0.07, *p* < 0.05) compared with younger children, but larger for boys (Coef. = 0.15, 95% CI 0.05–0.25, *p* < 0.05), the only child in the family (Coef. = 0.26, 95% CI 0.16–0.36, *p* < 0.05), those living in central areas (Coef. = 0.15, 95% CI 0.02–0.28, *p* < 0.05), and those with uninsured caregivers (Coef. = 0.35, 95% CI 0.05–0.65, *p* < 0.05). Among chronic disease patients and the elderly, those living in urban areas and central areas tended to suffer from a lower economic burden for ILI (*p* < 0.05) compared with rural and western residents, and those with urban employee medical insurance had a higher economic burden for ILI than others covered by medical insurance for urban and rural residents.

## 5. Discussion 

To the best of our knowledge, this is the first study using a nationally representative sample from 10 provinces in China to investigate the economic burden of ILI among children, patients with chronic diseases, and the elderly. This study estimated the prevalence of self-reported ILI, ILI-related healthcare-seeking behaviors, the economic burden of ILI, and its influencing factors among children, chronic disease patients, and the elderly in China.

A high prevalence of ILI (45.73% of children, 16.77% of chronic disease patients, and 12.70% of elderly people) was found among high-risk groups, especially among children. The results were similar to those reported in previous studies [16,17]. As for the healthcare-seeking behaviors, “Outpatient service only”, “OTC medication only”, and “Outpatient + OTC” were most frequently chosen, consistent with a previous study in which 86% influenza patients aged 60 years and above received ambulatory care only [18]. The elderly were more likely to receive inpatient services, indicating that older ILI patients were at higher risk of becoming severe cases [3]. In addition, a larger portion of chronic disease patients and the elderly did not receive any medical services after ILI, reflecting the problem that adult high-risk groups did not attach much importance to their health conditions after ILI, and a previous study also revealed that financial protection was the most important cause of not seeking care [36]. In this case, adequate public education on influenza disease, especially during the influenza season, may help raise people’s awareness of disease severity [37], and strengthened financial protection of health insurance can also help increase healthcare utilization, such as widened coverage of medical services and higher reimbursement rates [38].

After the occurrence of ILI, the economic burden of the elderly was the highest (CNY 1796), followed by children (CNY 1647) and chronic disease patients (CNY 951). It was found that outpatient service and OTC medication were most frequently chosen after ILI for the three groups, but the results of medical expenses demonstrated that once inpatient service was utilized in severe cases or patients with other complications, the medical burden of ILI would greatly increase in terms of both reimbursed expenses and self-paid expenses. Our estimates of ILI-related costs were higher than earlier estimates in China [16,17], similar to more recent estimates [22], but much lower than those reported in the United States, especially the lost productivity [13]. In the United States, studies found that indirect costs, including lost productivity from missed work days and lost lives, comprised a larger amount of influenza economic burden than hospitalization costs [12,13]. In the present study, direct medical and non-medical costs comprised a larger amount of economic burden of ILI, perhaps due to the fact that we only included lost productivity from missed work days in the indirect cost, and there are large gaps in labor costs between the two countries. Results derived from the present study on ILI economic burdens can help raise public awareness on the burdens and consequences of this important disease. Moreover, economic considerations are essential to effectively guide policy-making for influenza vaccination [39], so the direct and indirect economic costs of influenza at the national level are crucial to support national-level decision-making on the introduction of influenza vaccination as a public health project, complementary vaccination strategies, and/or expanding vaccination target groups [40].

There were several studies examining the incidence and economic burden of influenza at the hospital or provincial level in China. A population-based household survey conducted in 2007 in Guangdong Province found annual ILI incidence of 49.87% among children aged 1–4 years and 2.99% among the elderly aged >60 years, and the mean medical cost of one episode was CNY 172.5 for residents of all age groups [16]. A prospective study conducted in the 2011–2012 season among children <5 years in Suzhou reported that the mean direct and indirect costs per episode of influenza were CNY 777.4 for outpatient clinics and CNY 848.0 for emergency departments [19]. These results were similar to the ILI incidence and economic costs reported in the present study. As a rough estimate, the overall annual economic burden of ILI was about CNY 61.9 billion for children, CNY 25.0 billion for chronic disease patients, and CNY 38.0 billion for the elderly, based on the population size recorded in statistical yearbooks [27,35,41].

As for the co-payment of medical costs, the self-payment ratio and self-paid expenses were higher among children than chronic disease patients and the elderly due to the design of insurance schemes [42]. Moreover, compared with children and chronic disease patients, the elderly had a lower probability of catching ILI, but a higher economic burden after catching ILI. A previous study also found that young children and the elderly accounted for over 70% of the economic burden of influenza-associated hospitalizations in Jingzhou, China [22], indicating the importance of targeted interventions such as financed or reimbursed influenza vaccination programs [13], early treatment of ILI [43], and a higher reimbursement rate for high-risk groups [38], especially elderly people with less income and a larger economic burden after ILI, as well as children with higher prevalence of ILI and a higher self-payment ratio for medical expenses.

For children, the two-part model indicated that older children had a higher probability of ILI economic burden occurring, but smaller economic burden after ILI, revealing that older children had higher incidence of ILI, while younger children may have had more severe conditions after occurring ILI. In addition, boys had a higher probability of an ILI economic burden occurring and a larger economic burden after ILI, which may be related to boys’ lifestyles and potential son preference [44]. The economic burden after ILI was also larger for the only child in the family, as parents may switch investment from exclusively one child to others if they have more than one child [45]. As for adults, it was found that younger respondents who developed chronic diseases at an earlier age tended to have a higher probability of having an ILI economic burden compared with other chronic disease patients aged less than 60 years. Patients with a higher household income and adults with urban employee insurance reported larger economic costs after ILI in the three groups, but no significant difference was observed in the probability analysis (Part 1).

Regional or rural–urban differences were highlighted in the two-part analysis. For children, those living in central and eastern areas had higher probability of an ILI economic burden occurring, and those in central areas also had a higher economic burden after ILI. This was in line with previous findings on the economic burden of children with asthma (another respiratory disease) in China [46], which indicated that patients aged 0–14 years in central China had the highest use rate of medications, antibiotics, hematological tests, and chest X-rays. Reducing the unnecessary use of antibiotics and tests may help reduce young patients’ ILI economic burden in western areas [46]. In comparison, chronic disease patients and the elderly living in western or rural areas tended to suffer from a higher economic burden after ILI. Given the lower lost productivity and direct expenditures in less-developed areas, it was expected that rural and western residents might have smaller economic burdens for ILI than urban or eastern residents [16]. However, a higher economic burden was observed among rural and western adult residents in this population-based survey, and this might be due to the higher hospitalization rate in rural (23.04% for rural and 8.25% for urban) and western (23.03% for western, 14.85% for central, and 6.75% for eastern) patients in this study. This revealed the substantial regional and rural–urban gaps in Chinese adult patients concerning ILI economic burden, especially the hospitalization burden, and joint efforts are needed to reduce the gap and strengthen the financial protection for rural and western residents.

The present study also had a few limitations. First, the economic burden was collected from ILI cases instead of confirmed influenza cases. As recommended by the WHO, ILI sentinel surveillance data can be used to estimate the disease burden of influenza for specific risk groups [47,48,49], which is less specific but sensitive and rapid than laboratory surveillance [50]. Therefore, ILI cases were used, given that nationwide laboratory surveillance was not available, and a population-based study could offer higher population representativeness. Second, part of the elderly surveyed in this study were recruited from community health centers, which may have resulted in selection bias. Nevertheless, given the high prevalence of chronic diseases among Chinese elderly [51], and the fact that community centers mainly provide primary care, the bias could be reduced. Third, self-reported responses may be subject to recalling bias, and self-reported data may be affected by the severity of flu. To minimize the bias, we conducted a face-to-face on-site survey so that interviewees were more cautious about their answers, trained interviewers would help them recall expenditures if they had any difficulty, and we asked for respondents’ most recent influenza episode to minimize its effect. Fourth, in this cross-sectional survey, regression was performed only to show statistical correlations rather than inherent causal relations, which may have been subject to reverse causality. Fifth, in the regression, only a small number of respondents did not have any basic health insurance, making its coefficient not reliable enough, and future studies should be conducted to further detect the relationship between ILI economic burden and basic health insurance coverage. Despite these limitations, the nationally representative sample was large, with a diverse sociodemographic population, thus offering good generalizability for the three high-risk groups in China.

## 6. Conclusions

In conclusion, the prevalence of ILI was fairly high among the three priority groups in China, and outpatient service and/or OTC medication were most frequently chosen after ILI. The results highlighted the large economic burden of ILI, especially among two priority groups, including the elderly with less income and a larger economic burden after ILI, and children with a higher prevalence of ILI and a higher self-payment ratio for medical expenses. The financial burden of ILI on households can be viewed as an important healthcare issue, and it is time to adopt targeted interventions for children and the elderly, such as vaccinations and early treatment. At the same time, we should not ignore the regional and rural–urban differences of ILI economic burden in different priority groups. More importantly, this study can help decision-making on the introduction of influenza vaccination as a public health project in China for these high-risk groups by estimating the economic burden of influenza at the national level.

## Figures and Tables

**Table 1:** Summary of studies on the economic burden of influenza conducted in China.

Research Articles	Study Sites	Age Groups	Settings	Data Sources	Study Period	Composition of Economic Burden ^a^	Whether Healthcare-Seeking Behaviors Reported	Whether OTC Medication Costs Reported ^b^
Guo et al. (2011) [16]	Zhuhai and Zhaoqing cities in Guangdong province	All age groups	Population-based	Household survey	2006–2007	DM + I	No	No
Guo et al. (2012) [17]	Zhuhai city in Guangdong province	All age groups	Hospital-based (Outpatient)	Hospital diagnosis and telephone survey	2008–2009	DM + DnM	No	Yes
Chen et al. (2015) [18]	Shanghai city	The elderly aged ≥60 years	Hospital-based	Hospital diagnosis and follow-up survey	2009	DM	Yes	No
Wang et al. (2013) [19]	Suzhou city in Zhejiang province	Children aged <5 years	Hospital-based (Outpatient)	Hospital system and telephone survey	2011–2012	DM + DnM + I	Yes	No
Zhang et al. (2017) [20]	Suzhou city in Zhejiang province	Children aged <5 years	Hospital-based (Inpatient)	Hospital system	2005–2011	DM	No	No
Zhou et al. (2013) [21]	Three hospitals in Sichuan, Hunan and Shandong provinces	All age groups	Hospital-based (Inpatient)	Hospital system	2009–2011	DM	No	No
Yang et al. (2015) [22]	10 hospitals in 10 provinces and municipalities	All age groups	Hospital-based	Hospital diagnosis and telephone survey	2013–2014	DM + DnM + I	Yes	Yes

^a^ DM, direct medical burden; DnM, direct non-medical burden; I, indirect burden. ^b^ OTC, over-the-counter.

**Table 2:** Characteristics of 12,252 participants included in the analysis.

	Children Aged 6–59 Months			Chronic Disease Patients Aged 18–59 Years			Elderly Aged above 60 Years		
	n	ILI Cases, n (%)	*p*-Value	n	ILI Cases, n (%)	*p*-Value	n	ILI Cases, n (%)	*p*-Value
Total	6668	3049 (45.73)		1735	291 (16.77)		3849	489 (12.70)	
Age (years)			<0.01			<0.01			0.40
<2	3727	610 (16.37)		–	–		–	–	
3–5	2941	725 (24.65)		–	–		–	–	
18–39	–	–		72	24 (33.33)		–	–	
40–49	–	–		313	58 (18.53)		–	–	
50–59	–	–		1350	209 (15.48)		–	–	
60–69	–	–		–	–		2045	273 (13.35)	
70–79	–	–		–	–		1491	181 (12.14)	
≥80	–	–		–	–		313	35 (11.18)	
Gender			0.06			0.69			0.12
Female	3171	1412 (44.53)		1139	194 (17.03)		2334	281 (12.04)	
Male	3497	1637 (46.81)		596	97 (16.28)		1515	208 (13.73)	
The only child in the family			<0.01			–			–
Yes	3038	1320 (43.45)		–	–		–	–	
No	3630	1729 (47.63)		–	–		–	–	
Household monthly per capita income (CNY 1000) ^a^			0.62			0.24			<0.05
Mean (SD)	2.66 (2.76)	2.68 (2.69)		1.84 (2.41)	1.99 (2.31)		1.70 (1.61)	1.56 (1.86)	
Place of residence			0.33			0.94			<0.01
Rural	2814	1267 (45.02)		831	140 (16.85)		1742	255 (14.64)	
Urban	3854	1782 (46.24)		904	151 (16.7)		2107	234 (11.11)	
Region			<0.01			0.44			<0.01
Western	2219	793 (35.74)		602	99 (16.45)		1461	216 (14.78)	
Central	1932	1022 (52.90)		497	92 (18.51)		1035	136 (13.14)	
Eastern	2517	1234 (49.03)		636	100 (15.72)		1353	137 (10.13)	
Basic medical insurance type ^b^			0.15			0.20			<0.01
Medical insurance for urban and rural residents	4081	1842 (45.14)		1212	193 (15.92)		2551	356 (13.96)	
Urban employee medical insurance	2380	1121 (47.10)		492	90 (18.29)		1189	118 (9.92)	
Without basic medical insurance	207	86 (41.55)		31	8 (25.81)		109	15 (13.76)	
Self-reported health status			<0.01			<0.01			<0.01
Good	5812	2536 (43.63)		596	74 (12.42)		1578	141 (8.94)	
Fair or poor	856	513 (59.93)		1139	217 (19.05)		2271	348 (15.32)	

^a^ CNY 1 = USD 0.144 on 13 August 2020. ^b^ Basic medical insurance type in column refers to adult respondents.

**Table 3:** Healthcare-seeking behaviors of 3829 participants after influenza-like illness.

	Children Aged 6–59 Months		Chronic Disease Patients Aged 18–59 Years		Elderly Aged above 60 Years	
	n	%	n	%	n	%
Total	3049	100	291	100	489	100
Healthcare-seeking behaviors						
Outpatient service only	1162	38.11	100	34.36	159	32.52
Inpatient service only	46	1.51	4	1.37	26	5.32
OTC medication only ^a^	316	10.36	69	23.71	123	25.15
Outpatient + Inpatient	172	5.64	10	3.44	40	8.18
Outpatient + OTC	1092	35.82	77	26.46	77	15.75
Inpatient + OTC	25	0.82	1	0.34	14	2.86
Outpatient + Inpatient + OTC	134	4.39	10	3.44	18	3.68
No treatment	102	3.35	20	6.87	32	6.54
Service types						
Outpatient service	2560	83.96	197	67.70	294	60.12
Inpatient service	377	12.36	25	8.59	98	20.04
OTC medication	1567	51.39	157	53.95	232	47.44

^a^ OTC, over-the-counter.

**Table 4:** The economic burden of influenza-like illness for 3829 participants.

	Children Aged 6–59 Months		Chronic Disease Patients Aged 18–59 Years		Elderly Aged above 60 Years	
	Mean	95% CI ^a^	Mean	95% CI	Mean	95% CI
Total direct medical cost (CNY) ^b,e^	1269	(1168, 1370)	721	(512, 930)	1544	(1200, 1888)
Outpatient service	644	(595, 694)	239	(184, 293)	384	(281, 488)
Inpatient service	523	(454, 593)	394	(192, 595)	1053	(763, 1343)
OTC medication ^f^	102	(93, 110)	89	(64, 113)	106	(75, 138)
Reimbursed direct medical cost (CNY) ^b^	272	(234, 310)	335	(194, 477)	766	(568, 963)
Outpatient service	88	(67, 108)	85	(54, 116)	153	(95, 210)
Inpatient service	179	(151, 206)	239	(102, 375)	605	(426, 784)
OTC medication	6	(4, 8)	11	(6, 17)	8	(5, 12)
Self-paid direct medical cost (CNY) ^b^	997	(919, 1074)	386	(295, 477)	778	(595, 961)
Outpatient service	556	(515, 597)	154	(114, 193)	232	(167, 296)
Inpatient service	345	(295, 395)	155	(75, 235)	448	(302, 595)
OTC medication	96	(88, 103)	77	(54, 101)	98	(67, 130)
Direct non-medical cost (CNY) ^c^	212	(186, 238)	102	(31, 172)	162	(68, 256)
Transportation	79	(70, 87)	50	(−8, 109)	21	(8, 34)
Nutrition/food	107	(93, 121)	34	(18, 49)	96	(12, 180)
Accommodation	19	(12, 27)	18	(−2, 38)	40	(3, 76)
Nursing-worker hiring	8	(−5, 21)	0	(0, 0)	5	(−2, 12)
Indirect cost ^d^	166	(152, 179)	92	(60, 125)	90	(57, 124)
Lost labor days of respondents’ families	2	(1.9, 2.1)	0.7	(0.4, 1.0)	2	(1.5, 2.6)
Lost labor days of the respondents	–	–	1.1	(0.8, 1.5)	0.9	(0.5, 1.3)
Total economic costs	1647	(1527, 1768)	951	(671, 1159)	1796	(1413, 2179)

^a^ CI, confidence interval. ^b^ Direct medical cost was adjusted for the provincial healthcare price index in the China Statistical Yearbook 2019. ^c^ Direct non-medical cost was adjusted for the provincial consumer price index in the China Statistical Yearbook 2019. ^d^ Indirect cost was calculated based on the per capita GDP of each province in the China Statistical Yearbook 2019. ^e^ CNY 1 = USD 0.144 on 13 August 2020. ^f^ OTC, over-the-counter.

**Table 5:** Two-part regression of total economic burden (in log form) for the three groups.

Factors ^a^	Children		Chronic Disease Patients		Elderly	
	Part 1 Coef. (95% CI ^b^)	Part 2 Coef. (95% CI)	Part 1 Coef. (95% CI)	Part 2 Coef. (95% CI)	Part 1 Coef. (95% CI)	Part 2 Coef. (95% CI)
Age (years)						
<2	Ref.	Ref.	–	–	–	–
3–5	0.37 * (0.31, 0.43)	−0.16 * (−0.26, −0.07)	–	–	–	–
18–39	–	–	Ref.	Ref.	–	–
40–49	–	–	−0.47 * (−0.82, −0.12)	−0.55 (−1.29, 0.18)	–	–
50–59	–	–	−0.59 * (−0.91, −0.28)	−0.53 (−1.19, 0.14)	–	–
60–69	–	–	–	–	Ref.	Ref.
70–79	–	–	–	–	−0.07 (−0.18, 0.05)	0.05 (−0.33, 0.43)
≥80	–	–	–	–	−0.14 (−0.35, 0.06)	0.26 (−0.49, 1.01)
Gender						
Female	Ref.	Ref.	Ref.	Ref.	Ref.	Ref.
Male	0.06 * (0.00, 0.12)	0.15 * (0.05, 0.25)	−0.02 (−0.18, 0.13)	−0.22 (−0.59, 0.16)	0.10 (−0.01, 0.21)	−0.37 (−0.74, 0.00)
The only child in the family	−0.05 (−0.11, 0.02)	0.26 * (0.16, 0.36)	–	–	–	–
Household monthly per capita income (CNY 1000) ^c^	−0.01 (−0.02, 0.01)	0.02 * (0.00, 0.04)	0.02 (−0.02, 0.05)	0.12 * (0.03, 0.20)	0.02 (−0.02, 0.06)	0.14 * (0.01, 0.26)
Place of residence						
Rural	Ref.	Ref.	Ref.	Ref.	Ref.	Ref.
Urban	−0.01 (−0.08, 0.06)	0.08 (−0.02, 0.19)	−0.05 (−0.21, 0.11)	−0.45 * (−0.85, −0.06)	−0.11 (−0.23, 0.00)	−0.65 * (−1.07, −0.23)
Region						
Western	Ref.	Ref.	Ref.	Ref.	Ref.	Ref.
Central	0.50 * (0.42, 0.58)	0.15 * (0.02, 0.28)	0.07 (−0.11, 0.25)	−0.52 * (−0.95, −0.08)	−0.12 (−0.25, 0.01)	−0.57 * (−1.00, −0.13)
Eastern	0.40 * (0.32, 0.48)	0.07 (−0.06, 0.20)	−0.06 (−0.24, 0.12)	−0.15 (−0.60, 0.29)	−0.25 * (−0.38, −0.12)	−0.19 (−0.66, 0.28)
Basic medical insurance type ^d^						
Medical insurance for urban and rural residents	Ref.	Ref.	Ref.	Ref.	Ref.	Ref.
Urban employee medical insurance	0.04 (−0.03, 0.11)	0.06 (−0.05, 0.17)	0.05 (−0.13, 0.24)	0.60 * (0.14, 1.07)	−0.21 * (−0.36, −0.07)	0.56 * (0.05, 1.07)
Without basic medical insurance	−0.16 (−0.34, 0.02)	0.35 * (0.05, 0.65)	0.28 (−0.22, 0.78)	−1.07 (−2.19, 0.06)	−0.15 (−0.48, 0.18)	0.86 (−0.33, 2.04)

* Significant at the 5% level. ^a^ The results were controlled for self-reported health status. ^b^ CI, confidence interval. ^c^ CNY 1 = USD 0.144 on 13 August 2020. ^d^ Basic medical insurance type in column refers to adult respondents.

## Data Availability

The data presented in this study are available on request from the corresponding author. The data are not publicly available due to some restrictions, and they are only available on reasonable request.

## Appendix A

**Table A1:** Two-part regression of total economic burden (in log form) for the three groups (reporting marginal effects of Part 1).

Factors ^a^	Children	Chronic Disease Patients	Elderly
	Coef. (95% CI ^b^)	Coef. (95% CI)	Coef. (95% CI)
Age (years)			
<2	Ref.	–	–
3–5	0.14 * (0.12, 0.16)	–	–
18–39	–	Ref.	–
40–49	–	−0.14 * (−0.26, −0.03)	–
50–59	–	−0.17 * (−0.28, −0.06)	–
60–69	–	–	Ref.
70–79	–	–	−0.01 (−0.03, 0.01)
≥80	–	–	−0.03 (−0.06, 0.01)
Gender			
Female	Ref.	Ref.	Ref.
Male	0.02 * (0.00, 0.05)	−0.01 (−0.04, 0.03)	0.02 (0.00, 0.04)
The only child in the family	−0.02 (−0.04, 0.01)	–	–
Household monthly per capita income (CNY 1000) ^c^	0.00 (−0.01, 0.00)	0.00 (0.00, 0.01)	0.00 (0.00, 0.01)
Place of residence			
Rural	Ref.	Ref.	Ref.
Urban	0.00 (−0.03, 0.02)	−0.01 (−0.05, 0.03)	−0.02 (−0.04, 0.00)
Region			
Western	Ref.	Ref.	Ref.
Central	0.19 * (0.16, 0.22)	0.02 (−0.03, 0.06)	−0.03 (−0.05, 0.00)
Eastern	0.15 * (0.12, 0.18)	=0.01 (−0.06, 0.03)	−0.05 * (−0.07, −0.02)
Basic medical insurance type ^d^			
Medical insurance for urban and rural residents	Ref.	Ref.	Ref.
Urban employee medical insurance	0.02 (−0.01, 0.04)	0.01 (−0.03, 0.06)	−0.04 * (−0.07, −0.01)
Without basic medical insurance	−0.06 (−0.13, 0.01)	0.07 (−0.05, 0.19)	−0.03 (−0.09, 0.04)

* Significant at the 5% level. ^a^ The results were controlled for self-reported health status. ^b^ CI, confidence interval. ^c^ CNY 1 = USD 0.144 on 13 August 2020. ^d^ Basic medical insurance type in column refers to adult respondents.

## Appendix B

**Table A2:** Tobit regression of total economic burden (in log form) for the three groups.

Factors ^a^	Children		Chronic Disease Patients		Elderly	
	Coef.	95% CI ^b^	Coef.	95% CI	Coef.	95% CI
Age (years)						
<2	Ref.		–	–	–	–
3–5	1.95 *	(1.60, 2.31)	–	–	–	–
18–39	–	–	Ref.		–	–
40–49	–	–	−4.17 *	(−7.12, −1.23)	–	–
50–59	–	–	−5.28 *	(−7.99, −2.57)	–	–
60–69	–	–	–	–	Ref.	
70–79	–	–	–	–	−0.64	(−1.72, 0.44)
≥80	–	–	–	–	−1.31	(−3.32, 0.70)
Gender						
Female	Ref.		Ref.		Ref.	
Male	0.44 *	(0.09, 0.79)	−0.25	(−1.57, 1.07)	0.88	(−0.17, 1.94)
The only child in the family	−0.12	(−0.48, 0.24)	–	–	–	–
Household monthly per capita income (CNY 1000) ^c^	−0.03	(−0.10, 0.04)	0.15	(−0.11, 0.42)	0.23	(−0.16, 0.61)
Place of residence						
Rural	Ref.		Ref.		Ref.	
Urban	0.00	(−0.37, 0.38)	−0.57	(−1.95, 0.81)	−1.25 *	(−2.38, −0.11)
Region						
Western	Ref.		Ref.		Ref.	
Central	2.88 *	(2.42, 3.33)	0.44	(−1.11, 1.99)	−1.35 *	(−2.61, −0.09)
Eastern	2.31 *	(1.86, 2.75)	−0.60	(−2.15, 0.95)	−2.47 *	(−3.75, −1.19)
Basic medical insurance type ^d^						
Medical insurance for urban and rural residents	Ref.		Ref.		Ref.	
Urban employee medical insurance	0.25	(−0.15, 0.64)	0.64	(−0.95, 2.23)	−1.91 *	(−3.32, −0.50)
Without basic medical insurance	−0.73	(−1.77, 0.30)	1.88	(−2.50, 6.26)	−1.25	(−4.46, 1.96)

* Significant at the 5% level. ^a^ The results were controlled for self-reported health status. ^b^ CI, confidence interval. ^c^ CNY 1 = USD 0.144 on 13 August 2020. ^d^ Basic medical insurance type in column refers to adult respondents.
